# Weight Loss with Sleeve Gastrectomy in Obese Type 2 Diabetes Mellitus: Impact on Cardiac Function

**DOI:** 10.1007/s11695-015-1748-x

**Published:** 2015-06-20

**Authors:** Melissa Leung, Mikey Xie, Ertugrul Durmush, Dominic Y. Leung, Vincent W. Wong

**Affiliations:** Department of Cardiology, Liverpool Hospital, Locked Bag 7103, Liverpool BC, Sydney 1871 Australia; University of New South Wales, Sydney, NSW Australia; Liverpool Diabetes Collaborative Research Unit, Ingham Institute, Liverpool, Sydney Australia; Life Weight Loss Centre, Liverpool, Sydney Australia

**Keywords:** Bariatric surgery, Type 2 diabetes mellitus, Cardiac function

## Abstract

**Background:**

Diabetic cardiomyopathy is an increasingly prevalent health issue, with no specific management options. We examined the impact of weight loss with sleeve gastrectomy on diabetic cardiomyopathy.

**Methods:**

Eight obese patients with type 2 diabetes undergoing sleeve gastrectomy had left ventricular (LV) systolic and diastolic function assessed by global longitudinal strain (GLS) and septal early diastolic velocity (e’) using echocardiography, before and 9 months after surgery.

**Results:**

Following surgery, mean weight loss was 28.0 ± 16 kg; body mass index (BMI) decreased from 44 ± 9 to 35 ± 6 kg/m^2^ (*p* < 0.001). Glycaemic control improved with glycated haemoglobin (HbA1c) improving from 9.2 % at baseline to 6.7 % at follow-up (*p* = 0.002), with a corresponding improvement in LV GLS from −13.2 ± 3.7 to −19.7 ± 2.2 % (*p* < 0.001), and LV ejection fraction from 60 ± 5 to 70 ± 4 % (*p* < 0.001). Improvement in GLS was associated with the amount of weight lost (*ρ* = 0.81, *p* = 0.015). LV septal e’ velocities increased, and LV filling pressures decreased after surgery.

**Conclusions:**

Weight loss with sleeve gastrectomy in obese patients with type 2 diabetes is effective in improving glycaemic control in subjects with type 2 diabetes and results in significant improvement in both systolic and diastolic myocardial function.

## Introduction

Obesity and type 2 diabetes mellitus (T2DM) are prevalent health issues in both the developed and developing worlds. Patients with obesity and T2DM have an increased risk of developing left ventricular (LV) dysfunction and heart failure, independent of other traditional vascular risk factors. While diabetic cardiomyopathy is increasingly being recognised, specific treatment is still elusive. Diabetic cardiomyopathy may range from LV diastolic dysfunction in the early stages to overt severe global systolic dysfunction in the advanced stages of this disease. Smaller observational studies have shown that improved glycaemic control can improve LV diastolic dysfunction and suggest that cardiac abnormalities are potentially reversible [1]. However, these studies have failed to show any impact on systolic function.

Recent advances in echocardiography have allowed for more sensitive and accurate assessments of even subtle impairments in LV diastolic and systolic dysfunction. Tissue Doppler imaging (TDI) is a well accepted technique to assess myocardial tissue velocities; the measurement of the early diastolic velocity, e’, in combination with early (E-wave) and late diastolic (A-wave) mitral inflow pulsed-wave Doppler velocities allows for reliable assessment of LV filling pressures and diastolic function [2]. Two-dimensional (2D) speckle-tracking strain echocardiography has demonstrated ability to detect subtle, preclinical impairments in systolic function that are undetectable by conventional imaging techniques, including LV ejection fraction (LVEF) and fractional shortening [3, 4]. This superior sensitivity is ideal for the monitoring of the effects of treatment over shorter time periods [5]. Both TDI and strain imaging have been shown to be invaluable in the evaluation of diabetic cardiomyopathy [4].

In recent years, bariatric surgery has evolved as a successful management option for weight and glycaemic control in obese patients with poorly controlled T2DM. Buchwald et al. [6] found that 82 % of patients had resolution of both clinical and laboratory manifestations of T2DM in the 2 years following surgery. Resolution and improvement of hypertension, dyslipidaemia and sleep apnoea has also been demonstrated [7, 8]. Bariatric surgery has beneficial effects on cardiac function in obese patients without diabetes, with early studies demonstrating improvements in E/A ratio, shortening of isovolumetric relaxation time, regression of LV hypertrophy by LV mass [9], and improvement in longitudinal LV strain and strain rate [10]. However, it is unclear if bariatric surgery alters the progression or even reverses myocardial abnormalities of diabetic cardiomyopathy.

Laparoscopic sleeve gastrectomy is a safe and accepted treatment for obesity that involves removal of the fundus and most of the antrum of the stomach, thereby creating a gastric tube or sleeve that restricts oral intake. This technique has become increasingly popular due to its relative technical simplicity, preservation of the pylorus, and avoidance of postoperative malabsorption [11–13]. Few studies have evaluated the impact on laparoscopic sleeve gastrectomy on LV function in patients with T2DM. We aimed to study the effects of laparoscopic sleeve gastrectomy on LV function using the aforementioned advanced echocardiographic techniques.

## Materials and Methods

### Patients

The study population comprised obese patients with T2DM who were recruited and referred from a collaborative local weight loss centre. Patients with type 1 diabetes, current pregnancy, valvular or coronary artery disease (CAD), severe hypertension (systolic pressure >200 mmHg and diastolic pressure >120 mmHg at rest), left bundle branch block or non-sinus rhythm were excluded. Significant coronary artery disease was excluded by either coronary angiography or by stress echocardiography performed within 2 months of the study. All patients gave written informed consent. The study was conducted in accordance with National Health and Medical Research Council (NHMRC) guidelines and was approved by the hospital Human Research Ethics Committee.

All patients were assessed at study entry. Patients’ age, height, weight, waist and hip circumference, Medical Research Council (MRC) dyspnoea scale, cardiac risk factors, duration of DM, medications and the presence of macrovascular and microvascular complications were recorded. Metabolic data including glycated haemoglobin (HbA1c), serum creatinine, estimated glomerular filtration rate (eGFR), total cholesterol, low-density lipoprotein, high-density lipoprotein, triglycerides and spot urinary albumin-to-creatinine ratio were measured.

### Echocardiographic Studies

All patients underwent 2D transthoracic echocardiography at baseline. This was performed on patients in the left lateral decubitus position using commercially available ultrasound equipment (M5S probe, Vivid E9, General Electric (GE) Medical Systems, Milwaukee, WI). Images were stored for offline analysis (EchoPAC, BT13, GE). Complete M-mode, 2D, colour, pulsed and continuous-wave Doppler examination was performed according to standard techniques at rest to assess chamber thickness, volumes and valvular morphology. Mitral inflow E and A wave velocities, mitral septal annulus e’ velocities and LV mass were measured.

**Fig. 1:**
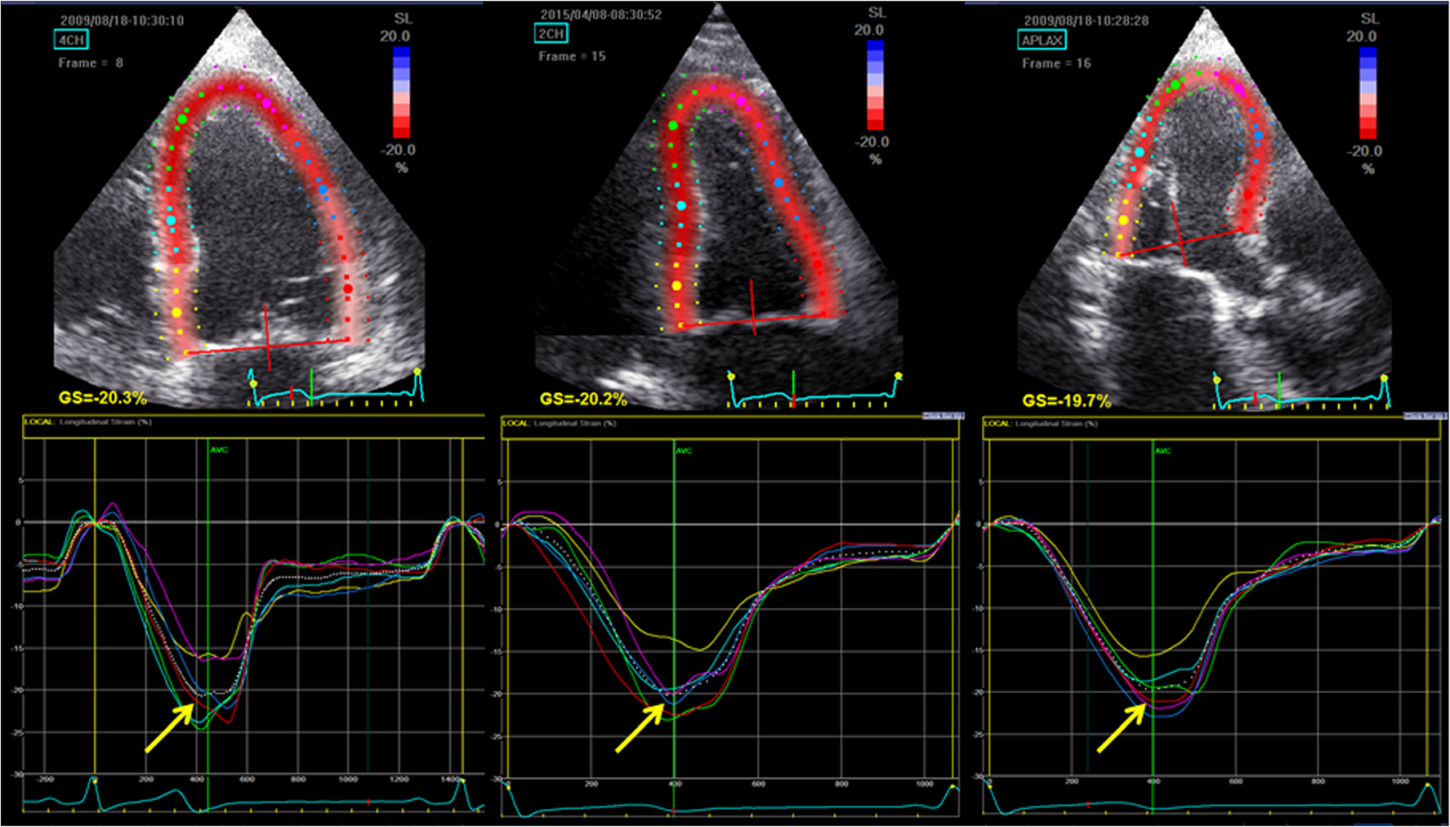
Example of calculation of global longitudinal strain (GLS) by 2D speckle tracking for the apical four-, two-, and three-chamber views. The strain throughout one cardiac cycle can be seen for each of the colour-coded LV segments (mean strain shown in *white*). In this example, the mean peak strains are *−20.3*, *−20.2*, and *−19.7 %* (indicated by the *arrows*), which occur during LV ejection. The calculated GLS is −20.1 %; a more negative strain indicates better systolic function. AVC aortic valve closure

Two-dimensional speckle-tracking analyses were used to measure LV global longitudinal strain (GLS). During these analyses, the endocardial border was manually traced at end-systole and the region of interest width was adjusted such that the entire myocardium was included. The software then tracks and accepts segments of good tracking quality and rejects poorly tracked segments. The observer is also able to manually accept and reject regions based on visual assessments of tracking quality. The mean GLS was calculated from the average of three global longitudinal strain curves of the three apical views (Fig. 1). All Doppler and 2D speckle-tracking echocardiographic measurements were calculated as averages of three representative cardiac cycles. Our inter- and intra-observer variabilities in measurement of LV GLS have been reported [14].

### Sleeve Gastrectomy and Weight Loss Program

Patients were assessed by a bariatric surgeon for suitability for laparoscopic sleeve gastrectomy. All patients adopted a preoperative very low calorie diet with an exercise program for 3 weeks supervised by an exercise physiologist, a dietician, and a psychologist. All patients underwent laparoscopic sleeve gastrectomy by a single surgeon. Postoperatively, diet included liquid diet for 3 weeks, then puree for 2 weeks, soft diet for 2 weeks, followed by progression to solid foods. After a 3-day stay in hospital, patients were seen postoperatively by the surgeon at 3-week, 3-month, 6-month and 1-year time points.

All patients underwent repeat clinical and biochemical assessment and echocardiographic studies 9 months after surgery.

### Statistical Analysis

Continuous variables were presented as mean ± 1 SD unless otherwise stated. Continuous variables before and after surgery were compared with the paired student’s *t* tests. Categorical variables were compared with the chi-square test. Linear regression was performed to identify relationship between improvement in GLS and e’ with changes in HbA1c with follow-up with Pearson’s or Spearman’s correlation coefficients (*ρ*). A two-sided *p* value <0.05 was considered significant. Statistical analyses were performed using STATA v12 (STATA Corporation, College Station, TX).

## Results

### Clinical and Metabolic Characteristics

Eight patients, two men and six women, with a mean age of 56 ± 7 years, were included. The baseline clinical and metabolic characteristics are presented in Table 1. Six patients (75 %) had history of hypertension, two were smokers and seven had dyslipidaemia. Two patients had peripheral vascular disease, two patients had diabetic retinopathy, five had peripheral neuropathy and five had micro-albuminuria.

**Table 1:** Clinical and metabolic characteristics of patients

Characteristic	Baseline (*n* = 8)	Follow-up (*n* = 8)	*p* value
Clinical			
Weight (kg)	126 ± 38	98 ± 24	0.002
Body mass index (BMI), kg/m^2^	44 ± 9	35 ± 6	<0.001
Body surface area (BSA), m^2^	2.40 ± 0.44	2.11 ± 0.33	<0.001
Waist circumference (cm)	128 ± 22	116 ± 11	0.160
Hip circumference (cm)	137 ± 18	120 ± 13	0.001
Waist-to-hip ratio (WHR)	0.94 ± 0.13	0.97 ± 0.06	0.407
Systolic BP (mmHg), mean ± SD	124 ± 19	131 ± 12	0.220
Diastolic BP (mmHg), mean ± SD	72 ± 12	79 ± 10	0.137
Metabolic characteristics			
HbA1c, %	9.2 ± 2.0 %	6.7 ± 1.3 %	0.002
Total cholesterol (mmol/L)	5.1 ± 1.7	4.5 ± 1.5	0.237
LDL cholesterol (mmol/L)	2.8 ± 1.4	2.5 ± 1.1	0.397
HDL cholesterol (mmol/L)	1.0 ± 0.2	1.2 ± 0.2	0.019
Triglycerides (mmol/L)	2.8 ± 1.2	1.8 ± 0.92	0.079
C-reactive protein (ng/mL)	11.5 ± 6.2	8.3 ± 7.8	0.134
Medications			
Aspirin or clopidogrel, *n* (%)	2 (25 %)	0 (0 %)	
ACE inhibitor or ARB, *n* (%)	7 (88 %)	5 (63 %)	0.375
Calcium channel blocker, *n* (%)	3 (38 %)	0 (0 %)	
Beta-blocker, *n* (%)	0 (0 %)	0 (0 %)	
Diuretic, *n* (%)	2 (25 %)	0 (0 %)	
Spironolactone, *n* (%)	0 (0 %)	0 (0 %)	
Statin, *n* (%)	4 (50 %)	5 (63 %)	0.143
Fibrate, *n* (%)	0 (0 %)	0 (0 %)	
Fish oil, *n* (%)	0 (0 %)	0 (0 %)	
Sulfonylurea, *n* (%)	3 (38 %)	3 (38 %)	1.000
Biguanide, *n* (%)	7 (88 %)	6 (75 %)	0.250
Thiazolidinedione, *n* (%)	1 (13 %)	0 (0 %)	
DPP4 inhibitor, *n* (%)	1 (13 %)	0 (0 %)	
Insulin, *n* (%)	5 (50 %)	1 (13 %)	1.000

**Fig. 2:**
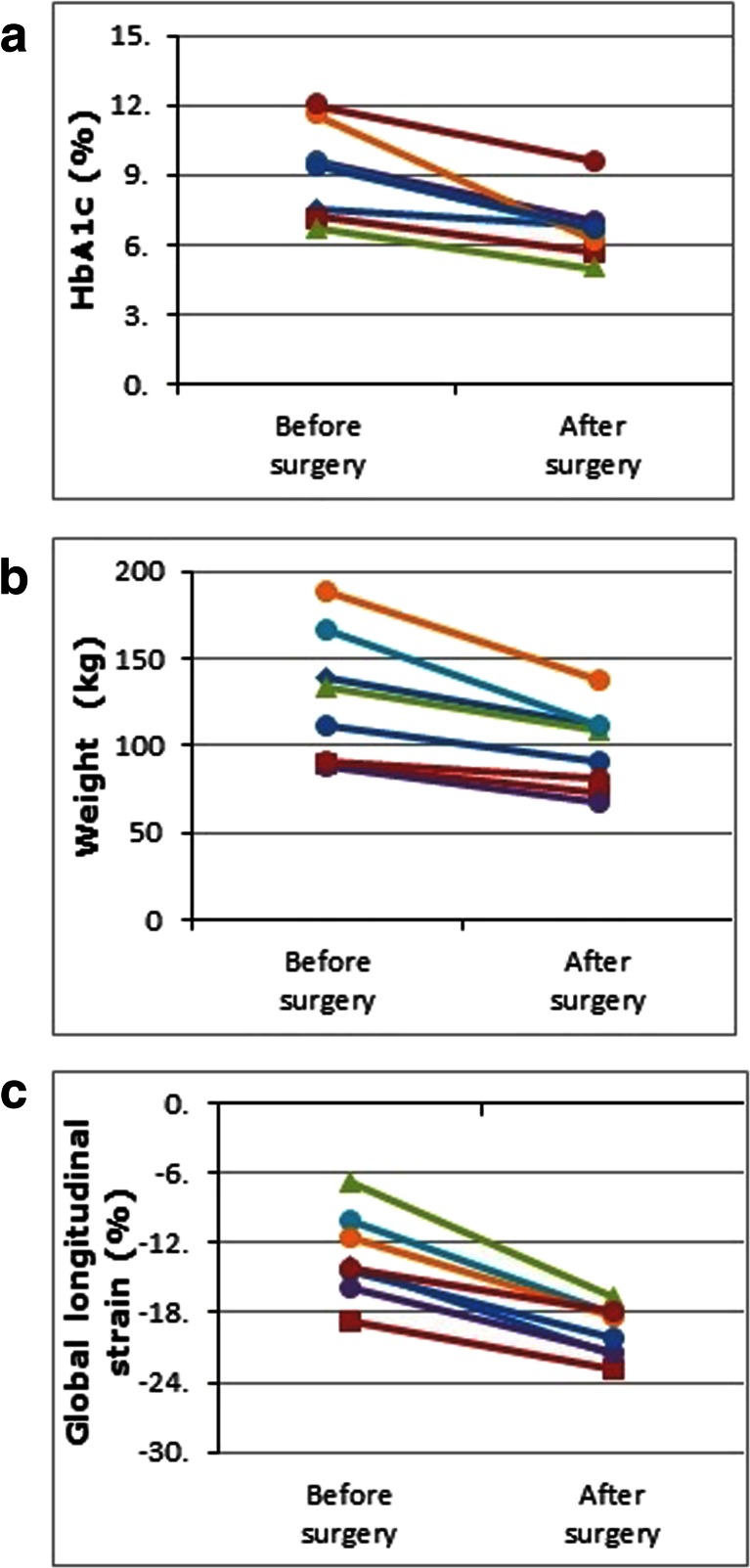
Improvements of the studied patients from baseline to follow-up in glycated haemoglobin HbA1c (panel **a**), weight (panel **b**) and global longitudinal strain (panel **c**)

Mean excess weight loss preoperatively was 4 % and from surgery until 9-month follow-up was 47 %. Surgery resulted in a significant decrease in body weight, body mass index and body surface area following surgery (Fig. 2). However, there was no significant change in the waist-to-hip ratio or blood pressure with surgery (Table 1).

Insulin requirements were reduced by sleeve gastrectomy. Five patients required insulin therapy prior to surgery, but only one patient remained on insulin at follow-up. Patients took an average of five different medications preoperatively and only three postoperatively (*p* = 0.008).

There was a significant decrease in HbA1c after surgery (Table 1). While the total cholesterol remained unchanged, there was a significant increase in HDL cholesterol.

### Echocardiographic Parameters

The echocardiographic data for the patients at baseline and follow-up are presented in Table 2. There was a significant improvement in LV systolic function as measured by LVEF and LV mean GLS after surgery, with all the patients demonstrating better GLS at follow-up (Fig. 2). Improvement in GLS showed a significant correlation with the amount of weight loss (*ρ* = 0.81, *p* = 0.015). However, there was no correlation between improvement in GLS and the decrease in HbA1c (*ρ* = 0.31, *p* = 0.456).

**Table 2:** Baseline and follow-up values of echocardiographic characteristics of the study group

Characteristic (mean ± SD)	Baseline *n* = 8	Follow-up *n* = 8	*p* value
LV mass index, g/m^2^	86 ± 30	77 ± 6	0.297
LVEF, %	60 ± 5	70 ± 4	<0.001
E/e’	12 ± 4	9 ± 3	0.028
E	71 ± 14	73 ± 10	0.787
A	79 ± 20	81 ± 18	0.641
E/A	0.93 ± 0.3	0.93 ± 0.3	1.000
Septal peak early diastolic velocity (e’), cm/s	6 ± 1	8 ± 2	0.015
LV global mean peak longitudinal systolic strain, %	−13.2 ± 3.7	−19.7 ± 2.2	<0.001

There was also a significant improvement in LV diastolic function measured by the septal e’ velocities and a reduction in LV filling pressures measured by the E/e’. There was no significant change in LV mass index. There were borderline correlations between the septal e’ velocities at follow-up with the final HbA1c (*r* = −0.66, *p* = 0.07) and with the amount of weight loss (*r* = 0.62, *p* = 0.1). Septal E/e’ ratio at follow-up was correlated with final HbA1c (*r* = 0.88, *p* = 0.004) and with the amount of weight loss (*r* = −0.69, *p* = 0.06).

### Other Health Outcomes

Patients reported improvements in symptoms of shortness of breath as measured by the MRC dyspnea scale (Table 3). All patients reported grade 1 on the MRC dyspnoea scale on follow-up. There were no postoperative complications such as infection, staple line leaks, or bleeding. None of the patients suffered any adverse cardiovascular events perioperatively or during the follow-up period.

**Table 3:** MRC dyspnoea grade

Characteristic	Before (*n* = 8)	After (*n* = 8)	*p* value
MRC dyspnea grade			
Grade 1	0 (0 %)	8 (100 %)	0.008
Grade 2	6 (75 %)	0 (0 %)	
Grade 3	2 (25 %)	0 (0 %)	
Grade 4	0 (0 %)	0 (0 %)	

## Discussion

Obese patients with T2DM have impairment of LV systolic and diastolic function. In this study, despite a normal LV ejection fraction of 60 % at baseline, our patients had significant impairment of LV systolic function as evident by a GLS of only −13 %. They also had impaired LV diastolic function with mean e’ velocities of 6 cm/s despite a mean patient age of only 56 years. LV filling pressures estimated by the E/e’ ratio were elevated in our patients. Sleeve gastrectomy led to significant weight loss as well as improvement in glycaemic control, but our study is novel in showing that this also resulted in significant improvement in both LV systolic and diastolic functions and a decrease in LV filling pressures in obese patients with T2DM. The more weight the patients lost after surgery, the larger the improvement in LV systolic function was. The improvement in LV systolic function was independent of the decrease in HbA1c with surgery. The lower the final HbA1c was and the more weight patients lost, the better their LV diastolic function at follow-up was. Similarly, the LV filling pressures were lower with lower final HbA1c or if there was larger amount of weight loss. Sleeve gastrectomy, combined with diet and lifestyle measures, resulted in significant weight loss and was associated with proportional improvements in both LV systolic and diastolic functions and a decrease in LV filling pressures.

Improvements in GLS following surgery have previously been shown in two non-randomised studies on obese patients without T2DM, but those studies did not demonstrate any changes in LVEF or E/e’ ratio [10, 15]. These studies included patients undergoing varying different types of bariatric surgery, varying durations of echocardiographic follow-up postoperatively (from 3 months onwards), and used less sensitive echocardiographic methods.

Other studies have also demonstrated improvements in the E/A ratio in obese patients without T2DM after bariatric surgery including sleeve gastrectomy and Roux-en-Y gastric bypass [16]. The current study demonstrated improvement in LV diastolic function without any changes in E/A ratio. E/A ratio is a very unreliable measure of LV diastolic function and filling pressures and should not be interpreted in isolation. The present study demonstrated improvements in septal e’ velocities which are a more reliable and less load-dependent measure of LV diastolic function, as well as an associated fall in LV filling pressures following sleeve gastrectomy.

Reduction in LV mass has previously been shown in various studies. The meta-analysis by Cuspidi et al. [16] combined data from 23 studies on the impacts of bariatric surgery on cardiac structure and function in non-diabetic patients and demonstrated significant changes in LV mass. The inability to demonstrate any decrease in LV mass in this study despite a moderate reduction of weight following surgery perhaps relates to the small sample size of the study as well as the relatively short duration of follow-up as detectable regression in LV mass may take longer than 9 months. The follow-up durations of the studies included in the meta-analysis by Cuspidi et al. were much longer than 9 months.

## Limitations

One of the main limitations of the study was the small sample size and that no control group was included for this study. The patients were followed up for 9 months following surgery, and a longer follow-up period could see further improvement in the patients’ glycaemic control and myocardial function.

## Conclusions

As far as we know, this is the first study to demonstrate that weight loss with sleeve gastrectomy is effective in improving glycaemic control in subjects with type 2 diabetes and results in significant and proportional improvement in both LV systolic and diastolic myocardial functions. While further research and larger studies are necessary to validate these findings, our study suggested that bariatric surgery not only has an important role in the management of obese patients with type 2 diabetes, it may also alter their progression of diabetic cardiomyopathy.

## References

[1] Von Bibra H, Hansen A, Dounis V (2004). Augmented metabolic control improves myocardial diastolic function and perfusion in patients with non-insulin dependent diabetes. Heart.

[2] Ommen S, Nishimura R, Appleton C (2000). Clinical utility of Doppler echocardiography and tissue Doppler imaging in the estimation of left ventricular filling pressures: a comparative simultaneous Doppler-catheterization study. Circulation.

[3] Ernande L, Rietzschel ER, Bergerot C (2010). Impaired myocardial radial function in asymptomatic patients with type 2 diabetes mellitus: a speckle-tracking imaging study. J Am Soc Echocardiogr.

[4] Ng AC, Delgado V, Bertini M (2009). Findings from left ventricular strain and strain rate imaging in asymptomatic patients with type 2 diabetes mellitus. Am J Cardiol.

[5] Sutherland GR, Di Salvo G, Claus P (2004). Strain and strain rate imaging: a new clinical approach to quantifying regional myocardial function. J Am Soc Echocardiogr.

[6] Buchwald H, Estok R, Fahrbach K, et al. Weight and type 2 diabetes after bariatric surgery: systematic review and meta-analysis. Am J Med. 2009;122:248–56. 10.1016/j.amjmed.2008.09.041 19272486

[7] Buchwald H, Avidor Y, Braunwald E (2004). Bariatric surgery: a systematic review and meta-analysis. JAMA.

[8] Sjöström L, Lindroos A-K, Peltonen M (2004). Lifestyle, diabetes, and cardiovascular risk factors 10 years after bariatric surgery. N Engl J Med.

[9] Ashrafian H, le Roux CW, Darzi A (2008). Effects of bariatric surgery on cardiovascular function. Circulation.

[10] Koshino Y, Villarraga HR, Somers VK (2013). Changes in myocardial mechanics in patients with obesity following major weight loss after bariatric surgery. Obesity.

[11] Kehagias I, Karamanakos SN, Argentou M (2011). Randomized clinical trial of laparoscopic Roux-en-Y gastric bypass versus laparoscopic sleeve gastrectomy for the management of patients with BMI <50 kg/m2. Obes Surg.

[12] Deitel M, Gagner M, Erickson AL (2011). Third International Summit: current status of sleeve gastrectomy. Surg Obesity Related Diseases: Off J Am Soc Bariatric Surg.

[13] D'Hondt M, Vanneste S, Pottel H (2011). Laparoscopic sleeve gastrectomy as a single-stage procedure for the treatment of morbid obesity and the resulting quality of life, resolution of comorbidities, food tolerance, and 6-year weight loss. Surg Endosc.

[14] Leung M, Juergens CP, Lo ST (2014). Evaluation of coronary microvascular function by left ventricular contractile reserve with low-dose dobutamine echocardiography. EuroIntervention.

[15] Di Bello V, Santini F, Di Cori A (2007). Effects of bariatric surgery on early myocardial alterations in adult severely obese subjects. Cardiology.

[16] Cuspidi C, Rescaldani M, Tadic M (2013). Effects of bariatric surgery on cardiac structure and function: a systematic review and meta-analysis. Am J Hypertens.

